# iTRAQ-Based Identification of Proteins Related to Lignin Synthesis in the Pear Pollinated with Pollen from Different Varieties

**DOI:** 10.3390/molecules23030548

**Published:** 2018-03-01

**Authors:** Shumei Li, Xueqiang Su, Qing Jin, Guohui Li, Yanming Sun, Muhammad Abdullah, Yongping Cai, Yi Lin

**Affiliations:** School of Life Science, Anhui Agricultural University, Hefei 230036, Anhui, China; lishushumei@163.com (S.L.); 15710006@ahau.edu.cn (X.S.); qingjin@ahau.edu.cn (Q.J.); zhuzhu3278@sina.com (G.L.); 18225863536@163.com (Y.S.); abdullahpadana@hotmail.com (M.A.)

**Keywords:** Dangshan Su pear, iTRAQ, different pollinations, proteomic, lignin, phenylpropanoid pathway

## Abstract

Most pears in Anhui Province are a kind of self-incompatible fruit whose quality is strongly influenced by the male pollen. The proteomic variation of Dangshan Su pollinated by different varieties was analysed using the isobaric tag for relative and absolute quantitation (iTRAQ) to investigate the effect of pollination by different varieties on the pear lignin pathway. Among the 3980 proteins identified from the two samples, 139 proteins were identified as differentially expressed proteins (DEPs). Of these proteins, laccase-4 (LAC4), was found to be related with lignin synthesis, and β-glucosidase 15 (BGLU15) and peroxidase 47 (PER47) were involved in the phenylpropanoid pathway. Moreover, the lignin and stone cell contents were lower in DW (Dangshan Su pollinated by Wonhwang) than those in DJ (Dangshan Su pollinated by Jingbaili). The effect of pollination on the synthesis of lignin through the regulation of the expression of PER47, BGLU15 and LAC4 ultimately affects the formation of stone cells and the fruit quality. We report for the first time that different pollinations influence the protein expression profile in the Dangshan Su pear, and this result provides some new epididymal targets for regulating the synthesis of lignin, regulating the content of stone cells and improving the quality of the pears.

## 1. Introduction

*Pyrus bretschneideri cv*. Dangshan Su is a native species of China. Most pears are a kind of self-incompatible fruits with obvious xenia [1,2]. The fruit quality is affected to a great degree by pollination from different varieties [3]. The effects can be seen in such attributes as the fruit hardness and sugar, acid, vitamin C and stone cell content [4,5]. In recent years, due to inappropriate choices of pollination trees, the stone cell content and the degree of polymerization have sharply increased in pear pulp, ultimately affecting pear flavour and quality.

**Figure 1:**
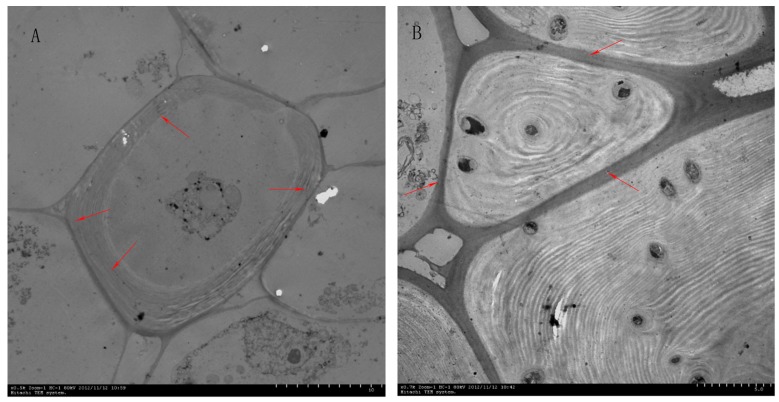
Lignin deposition during stone cell development. (**A**) Lignin was unevenly deposited along the primary wall as fine particles at the corner of the primary cell wall; (**B**) Lignin fully occupied the cell cavity. Note: the red arrows in picture A point to the lignin that deposited along the primary wall; the red arrows in picture B point to the stone cell.

Stone cells are a type of sclerenchyma cell formed by the secondary deposition of lignin on the primary walls of parenchyma cells [6]; thus, the formation of stone cells includes two important stages: synthesis and accumulation of lignin, followed by lignin deposition on the primary wall and secondary thickening. Therefore, the development of stone cells is closely related to the synthesis, transfer and deposition of lignin (Figure 1) [7]. The types of lignin in pear fruit are guaiacyl lignin (G-lignin) and syringyl lignin (S-lignin) [8].

Lignin biosynthesis follows the phenylpropane metabolism [9]. The synthesis of lignin in pear fruit starts with the deamination of phenylalanine to form cinnamic acid by the phenylalanine aminolytic enzyme, which includes a series of hydroxylation, methylation and oxidation reduction reactions. Finally, two major monomers for lignin biosynthesis are formed: coniferyl and sinapyl alcohol. These monomers then polymerize to form two types monolignol: S-lignin and G-lignin. A variety of enzymes are involved in the lignin biosynthesis process, such as phenylalanine ammonia-lyase (PAL), cinnamate-4-hydroxylase (C4H), coumarate 3-hydroxylase (C3H), caffeic acid/5-hydroxy-ferulic acid *O*-methyltransferase (COMT), ferulate-5-hydroxylase (F5H), 4-coumarate-CoA ligase (4CL), caffeoyl-CoA 3-*O*-methytransferase (CCoAOMT), cinnamoyl-CoA reductase (CCR), cinnamyl alcohol dehydrogenase (CAD), peroxidase (POD) and laccase (LAC) [10,11,12]. The expression of these enzymes affects the synthesis and content of lignin [13].

Our research group has studied the Dangshan Su pollinated by different pollen varieties and found that the pollination type affects the content of stone cells and lignin and the expression of microRNAs [5]. Proteomic analysis of Dangshan Su pears at different developmental stages has been reported [14], and a number of enzymes associated with lignin biosynthesis have been detected, including PAL, F5H, 4CL, CCR, CAD and POD. However, proteomic studies of Dangshan Su pollinated by different varieties have not been reported.

In previous studies, proteomic profiling has been shown to be a direct reflection of protein expression patterns that provides information about protein regulation and active pathways [15].

Over the past few decades, many proteomic platforms, such as two-dimensional gel electrophoresis and LC MS/MS, have been developed for the qualitative and quantitative characterization of protein mixtures. Currently, iTRAQ labelling technology presents unique advantages over other conventional proteomic techniques. The advantages of iTRAQ include allowing simultaneous comparison of protein profiles in multiple samples, providing information on peptide quantitation and identification, and being a relatively high-throughput process [16,17].

iTRAQ has been widely used for micro-organisms, fauna, and flora [18,19]. We selected iTRAQ technology to analyse the proteomic changes of Dangshan Su pollinated by different varieties to further explore the influence of different pollination varieties on the formation of lignin as well as the development of stone cells, which would lay a theoretical foundation for the improvement of stone cell content and lignin synthesis in Dangshan Su.

The pollen of Wonhwang (*Pyrus pyrifolia* Nakai.) and Jingbaili (*Pyrus ussuriensis* Maxim.) were used to fertilize Dangshan Su in Dangshan, Anhui Province, China. In the study, iTRAQ labelling technology was used to analyse the dynamic changes of proteins in Dangshan Su produced by different pollinations. These changes and an overall proteomic analysis were initiated to explore the potential effect of pollination on the Dangshan Su proteome. Our results set the theoretical foundation to improve fruit quality through the selection of optimal pollination varieties for Dangshan Su and provides the molecular basis for future improvement of fruit quality.

## 2. Results

### 2.1. Effect of Different Pollinations on Dangshan Su Stone Cell Content and Lignin Content

**Figure 2:**
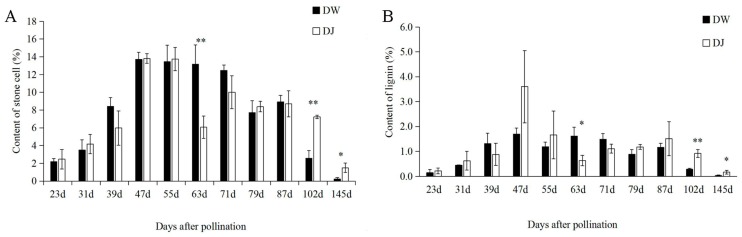
Stone cell content and lignin content during different developmental stages of the DJ and DW. (**A**) Content of stone cells; (**B**) Content of lignin. Note: * means significant difference; ** means very significant difference.

The variation trends of stone cell content during different developmental stages were the same between DW and DJ, with an initial increase up to the maximum followed by a decrease (Figure 2A). The stone cell content of DW increased to the maximum at 47 days after pollination (DAPs), followed by a decrease to reach the minimum at 145 DAPs. The stone cell content of DW was 0.24% at 145 DAPs. Two peaks of stone cell content in DJ appeared at 47 and 71 DAPs. The stone cell content of DJ was 1.04% at 145 DAPs. Since the content of stone cells in DJ was higher than that in DW, the quality of DW was considered better than DJ.

The content of lignin during different developmental stages was the same between DW and DJ, increasing to the maximum, followed by a decrease (Figure 2B). The lignin content of DW showed two peaks at 47 and 63 DAPs of 1.69% and 1.62%, respectively. The lignin content dipped to minimum of 0.03% at 145 DAPs. The lignin content of DJ increased to the maximum of 3.60% at 47 DAPs and dipped to the minimum of 0.11% at 145 DAPs. The content of lignin in DJ was higher than that in DW.

Through SPSS 18.0 bivariate analysis, the correlation coefficient of lignin and stone cell content was 0.958 ** and 0.853 ** in DW and DJ, respectively, indicating that the content of stone cells in Dangshan Su pollinated with different varieties was significantly affected by the changing of lignin content. This was in accordance with the results of Cai [20].

According to previous experiments, the synthesis of lignin occurs from 15 DAPs to 63 DAPs. In addition, the lignin content of DW and DJ reached a peak at 47 DAPs, with the lignin content in DJ being 2.1 times higher than that in DW. Thus, the 47 DAPs compose the critical period for lignin synthesis. Dangshan Su pollinated with different varieties were selected for further proteomic analysis at 47 DAPs because the difference in lignin content was larger at this point.

### 2.2. Protein Identification and Quantification

**Figure 3:**
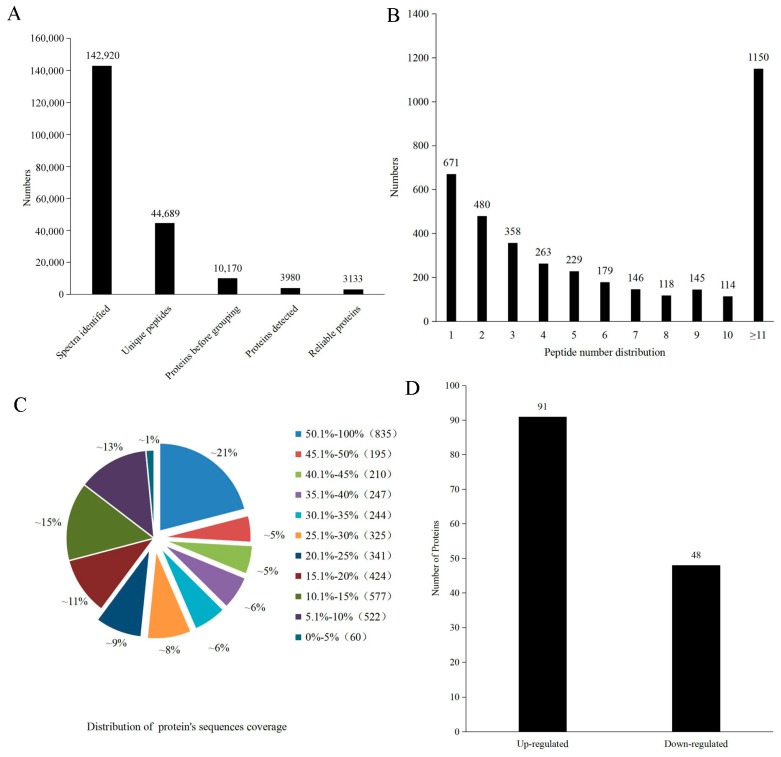
Basic information of iTRAQ output. (**A**) Proteome identification in pear fruit; (**B**) Number of peptides that matched proteins; (**C**) Coverage of the proteins by the identified peptides; (**D**) Number of differentially expressed proteins.

By the iTRAQ labelling and 2D LC-MS/MS method, a total of 142,920 spectra were acquired from pear fruit with different pollination varieties. After data filtering to eliminate low-scoring spectra, 44,689 unique spectra were matched to 44,689 unique peptides, and a total of 3980 proteins were identified from pear fruit with a >95% confidence level, along with 3133 reliable proteins (Figure 3A). Among the identified proteins, 671 proteins had one identified peptide, 480 had two, 1150 had more than 11, and the remainder had 3–10 (Figure 3B). The peptide sequence coverage was primarily more than 20% (Figure 3C). Because iTRAQ quantification underestimated the amount of real-fold change between two samples, proteins with a fold-change >1.2 or <0.83 in abundance were regarded as DEPs. Based on these criteria, 139 DEPs in the DW and DJ were selected for further analysis. Of these, 91 DEPs increased in the DW, and 48 DEPs decreased in the DW (Figure 3D).

### 2.3. Functional Analysis and Annotation of Differentially Expressed Proteins

**Figure 4:**
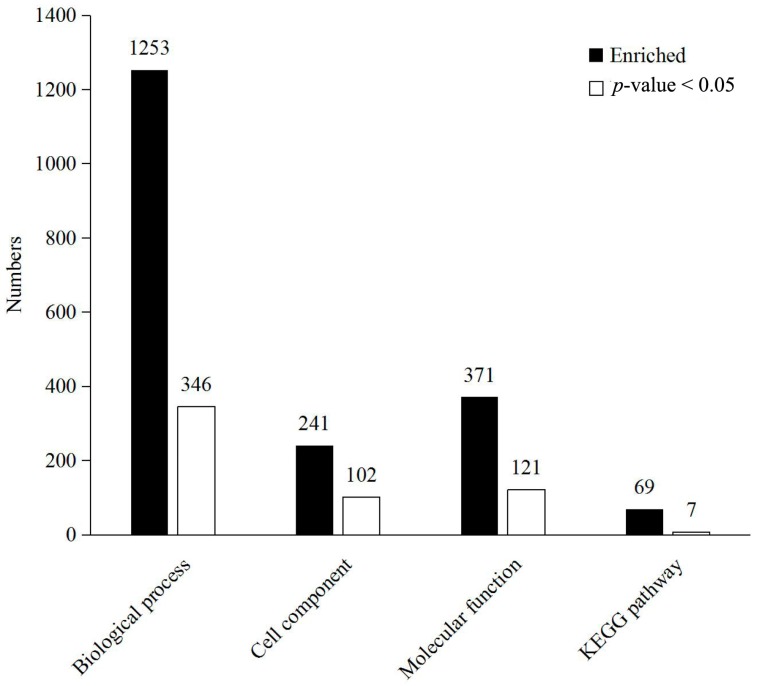
Bioinformatics analysis of 139 identified differentially expressed proteins. Biological process, cellular component, molecular function and KEGG were the 4 categories of functional analysis. Counts for each category represent the total terms in the database associated with the queried protein list. Terms with a *p*-value < 0.05 were considered significant.

The 139 DEPs were analysed using bioinformatics approaches in an effort to extract information relevant to the involved pathways. From analysis of all of the DEPs via gene ontology (GO) enrichment in the biological process, cellular component and molecular function, and Kyoto Encyclopedia of Genes and Genomes (KEGG) pathway enrichment, the results were 1235, 241, 371 and 69, respectively. In general, 346, 102, 121 and 7 terms were significantly enriched in the biological process, cellular component, molecular function and KEGG pathway, respectively (Figure 4). DEPs were annotated with categories simultaneously. For the biological process category, there were 121, 121 and 112 DEPs involved in biological processes, cellular components and molecular function, respectively.

**Figure 5:**
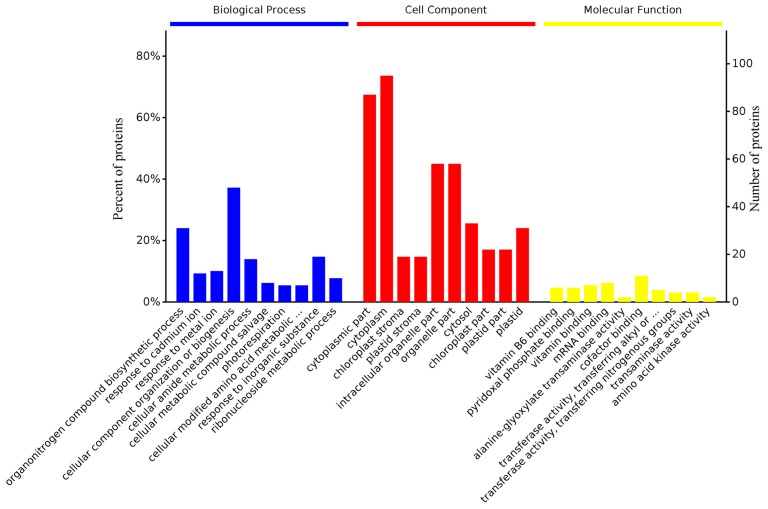
The top ten most significantly enriched terms through gene ontology hierarchy. Information on the percentage and number of involved proteins in a term is shown on the left and right *y*-axes.

Moreover, the top ten enriched terms by gene ontology hierarchy are depicted in Figure 5. Among these, the organonitrogen compound biosynthetic process, cytoplasmic components and vitamin B6 binding were the most representative terms in biological processes, cellular components and molecular function, respectively.

**Figure 6:**
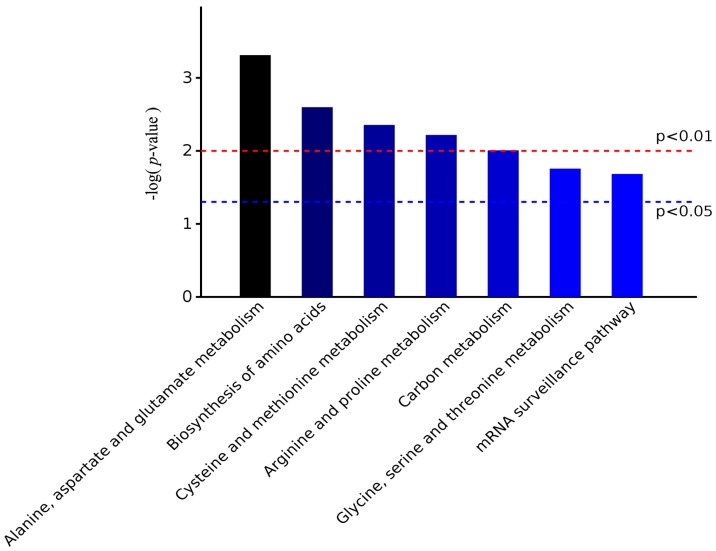
The significant entries according to the KEGG pathway enrichment analysis of differentially expressed proteins.

**Figure 7:**
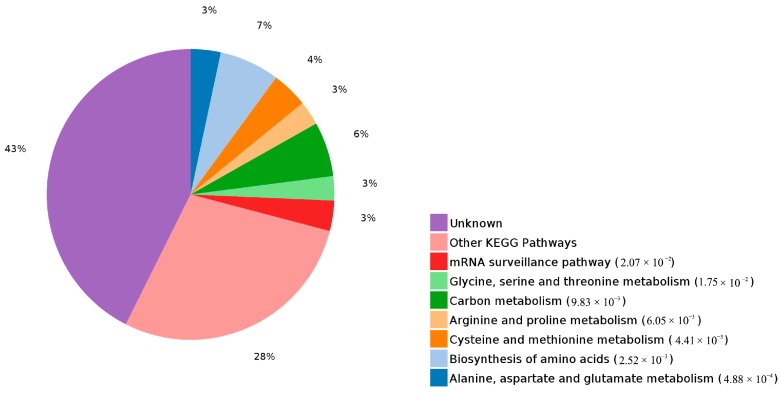
The significantly enriched KEGG pathways of differentially expressed proteins.

By the analysis of the significantly enriched terms in the KEGG pathway, five entries were identified as very significant, and seven entries were significant. The most significant terms were alanine, aspartate and glutamate metabolism, followed by biosynthesis of amino acids, all of which belonged to the amino acid metabolic pathway (Figure 6). Clustering analysis of DEPs by the KEGG pathway (Figure 7) revealed that the percent of alanine, aspartate and glutamate metabolism was 7%, which was the largest proportion of DEPs enriched in KEGG metabolism.

### 2.4. Functional Annotation of Differentially Expressed Proteins Involves in Lignin Biosynthesis

To explore the effect of pollination by different varieties on the proteins involved in lignin biosynthesis in pear fruit, the lignin biosynthetic process enriched in GO analysis and the phenylpropane metabolism enriched in KEGG analysis was further analysed.

The enriched terms by GO hierarchy (max. level 7) are depicted in Table S1. The lignin biosynthesis process was enriched with a *p*-value of 0.183. Among the DEPs, Pbr035962.1 (LAC4) was associated with the lignin biosynthesis process. The DEPs of KEGG enrichment analysis showed two DEPs enriched in phenylpropane metabolism, MSTRG11809.4 (BGLU15) and Pbr022326.1 (PER47). Thus, the DEPs in the pear fruit of trees pollinated by different varieties associated with the lignin biosynthesis process and phenylpropane metabolism were Pbr035962.1(LAC4), MSTRG11809.4 (BGLU15) and Pbr022326.1 (PER47) (Table 1).

In the analysis of reliable proteins, a total of 52 proteins were involved in phenylpropanoid biosynthesis (Table S2), including two differentially expressed proteins, MSTRG11809.4 (BGLU15) and Pbr022326.1 (PER47). According to analysis of the phenylpropanoid biosynthesis enzymes, several key enzymes related to the synthesis of lignin were identified in Dangshan Su pollinated by different varieties, including PAL (Pbr008363.1, Pbr005830.1), C3′H (MSTRG11837.4, Pbr020886.1, Pbr026583.1), C4H (Pbr017290.1, Pbr013141.1), F5H (Pbr022142.1, Pbr040547.1), 4CL (Pbr013445.1, Pbr024635.1, MSTRG13241.2, MSTRG4502.2), CCR (Pbr022405.1), CAD (Pbr026287.1, Pbr010181.1), OMT (Pbr013510.1, Pbr023162.1), CCoAOMT (Pbr034039.1, Pbr019305.1), and PER (Pbr010364.1, Pbr000691.1, Pbr000689.1, Pbr040489.1, Pbr027164.1, Pbr026235.1, Pbr035186.1, Pbr001738.1, Pbr011562.1, Pbr031894.1, MSTRG5854.1, Pbr010429.1, Pbr009308.1, MSTRG23544.1, Pbr022326.1, Pbr034800.1).

### 2.5. Protein-Protein Interaction Analysis of Differently Pollinated Dangshan Su

**Figure 8:**
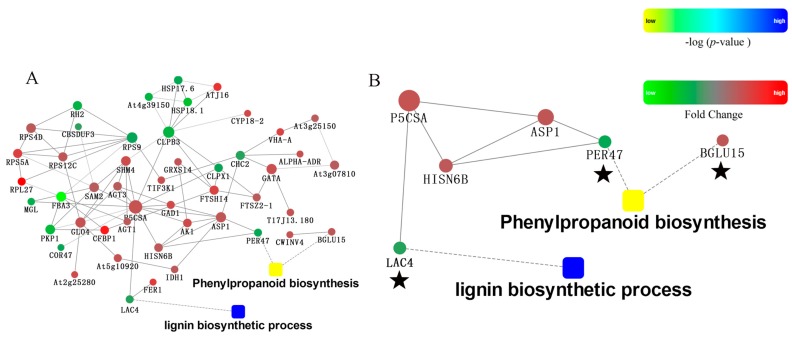
Protein-protein interactions were obtained using the string database. (**A**) Complete picture of PPI analysis; (**B**) Diagram of PPI analysis.

Through the protein-protein interaction (PPI) analysis of differentially expressed proteins (Figure 8A), MSTRG11809.4 (BGLU15) and Pbr022326.1 (PER47) were related to phenylpropanoid biosynthesis, and Pbr035962.1 (LAC4) was related to the lignin biosynthesis process. Pbr022326.1 (PER47) and Pbr035962.1 (LAC4) had an indirect interaction relationship through the connection of MSTRG471.1 (P5CSA) and MSTRG16606.2 (ASP1) or of MSTRG471.1 (P5CSA) and Pbr017805.1 (HISN6B) (Figure 8B). Although these predicted interaction networks need to be verified, they have provided a narrow pool of protein-protein interactions in Dangshan Su in response to pollination by different varieties for use in further investigations.

### 2.6. Verification of Differentially Expressed Genes by qRT-PCR

The fold change of MSTRG11809.4 (*PbBGLU15*), Pbr022326.1 (*PbPER47*) and Pbr035962.1 (*PbIRX12*) relative expression in DW compared to DJ was 1.0778, 18.0000 and 1.0282, respectively. The relative expression levels of these proteins in DW at 47 DAPs were up-regulated.

**Figure 9:**
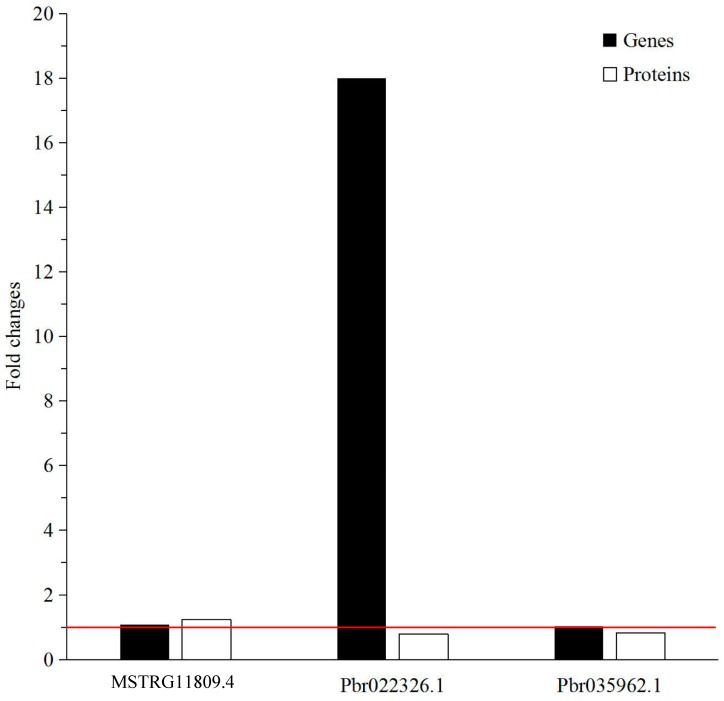
The expression fold changes at the mRNA and protein levels of three DEPs that were involved in phenylpropanoid biosynthesis and lignin formation. The red line represents a fold change equal to 1.

In this study, the protein-associated genes that were actually differentially expressed in differently pollinated pear fruit were verified. Through the verification of three DEPs by qRT-PCR, the relative expression of MSTRG11809.4 on the level of genes and proteins was coincident and up-regulated in DW. The relative expression of Pbr022326.1 and Pbr035962.1 on the level of genes and proteins did not match. Pbr022326.1 and Pbr035962.1 were up-regulated at the gene level and down-regulated at the protein level (Figure 9).

## 3. Discussion

Stone cells exist in pear fruit and are negatively correlated with the fruit quality [7]. In the detection of stone cells and lignin content in Dangshan Su fruit that had been pollinated with different varieties, the stone cell content of DW is found to be lower than that of DJ, and the lignin contents of DW and DJ are 0.03% and 0.11%, respectively. The quality of DW is better, so the Wonwhang is determined as one of the more appropriate pollination varieties for Dangshan Su.

Stone cell formation is a secondary cell wall-thickening process. Lignin, which is a polymer of phenolic compounds, is the main component of the cell wall secondary structure and is synthesized by the “phenylpropanoid biosynthesis” pathway.

**Figure 10:**
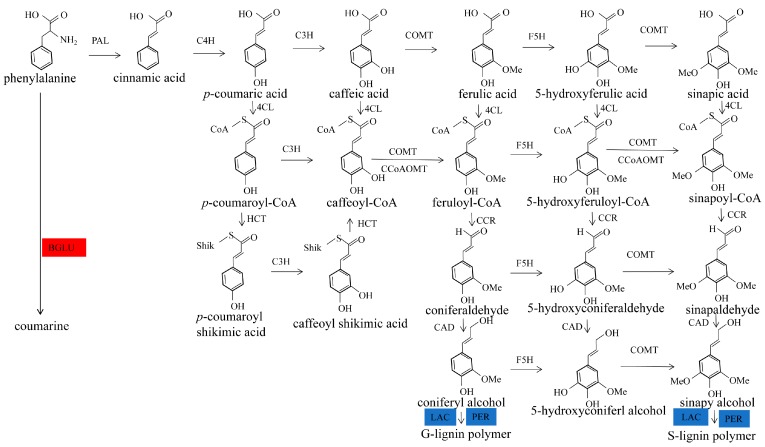
Differentially expressed proteins involved in lignin formation. Red boxes indicate up-regulated proteins in DW; blue boxes indicate proteins down-regulated in DJ.

According to the comparative proteomic analysis of Dangshan Su fruit pollinated by different varieties, a total of 3133 reliable proteins were identified from pear fruit, of which 139 proteins were differentially expressed. The 52 identified credible proteins were related to phenylpropanoid biosynthesis. Considering the bioinformatic analysis of the differentially expressed proteins, functional analysis indicated that Pbr035962.1 (LAC4) was related to the lignin biosynthetic process. Based on the analysis of the KEGG pathway, two DEPs, MSTRG11809.4 (BGLU15) and Pbr022326.1 (PER47), were enriched in phenylpropanoid biosynthesis. The differentially expressed proteins associated with lignin formation were MSTRG11809.4 (BGLU15), Pbr022326.1 (PER47) and Pbr035962.1 (LAC4) (Figure 10).

β-Glucosidase is not only related to phenylpropanoid biosynthesis, but also affects starch and sucrose metabolism (KO00500), converting insoluble cellulose into oligosaccharides. The primary wall is mainly composed of cellulose and pectin, and the secondary wall is the cell wall that accumulates at inner part of the primary wall after the cell stops growing. Lignin in plants is mainly distributed in the intercellular matrix, xylem tubular molecules and fibres at secondary walls, specific types of epidermal cells, and the secondary wall of sclerenchymatous cells [21,22,23]. Therefore, beta-glucosidase can affect the formation of lignin. The expression of β-glucosidase in DW was up-regulated, which reduced the formation of primary cell walls, resulting in the reduction of lignin content, which is coincident with the measurement of lignin content in DW being lower than that in DJ at 47 DAPs.

In the process of lignin synthesis, lignin monomers are polymerized by peroxidase (PER) and laccase (LAC) to form lignin polymers [24,25,26]. The lignin content at 47 DAPs in DW was lower than in DJ, and the expression of peroxidase and laccase was down-regulated in DW [8]. It is speculated that peroxidase and laccase play an important role in lignin biosynthesis, so the down-regulated expression of peroxidase and laccase in DW leads to its lower lignin content. Finally, the stone cell content in DW was lower and the quality of DW was better, so we propose that Wonhwang is the suitable pollination tree for Dangshan Su.

Pollination by different varieties affects the expression of PER and LAC that relate the lignin biosynthesis process, thereby affecting the content of lignin in pear fruit. Although BGLU is not involved in the lignin biosynthesis, it affects the synthesis of lignin indirectly by influencing the formation of cell walls. In brief, pollination of Dangshan Su by different varieties affected the expression of proteins related to lignin biosynthesis in fruits, thus affecting the synthesis of lignin and, ultimately, the content of stone cells and the quality of pear fruit.

In DW at 47 DAPs, the changes between gene levels and protein levels of some proteins were inconsistent. A number of studies have verified this finding by statistical methods [27]. (1) Protein post-translational modifications, such as phosphorylation, glycosylation, ubiquitination, etc., may affect protein secretion and degradation [28]; (2) Studies have shown that the stability of mRNA after gene transcription is related to its specific nucleotide sequence of three ’UTR and the corresponding binding proteins [29]; (3) The effects of post transcriptional regulation on different genes are inconsistent, thus leading to the different expression levels of various genes.

## 4. Materials and Methods

### 4.1. Processing and Collection of Pear Fruits

Fruits were obtained from 50-year-old pear trees grown in Dangshan, Anhui Province, China. In April, twenty robust and healthy trees managed in a consistent manner were selected as mother trees. The Wonhwang (*Pyrus pyrifolia* Nakai.) and Jingbaili (*Pyrus ussuriensis* Maxim.) were selected as father trees, and pollen was collected from the buds. Buds on the short branches of Dangshan Su with similar developmental stages and sizes were selected from the mid-crown area on the south side of each tree, from which the stamen was removed, and the bud was fertilized with pollen from *P. pyrifolia cv.* Wonhwang or *P. ussuriensis cv.* Jingbaili. The buds were covered with bags for seven days after pollination. Two fruits were kept on each short branch. Fruits were collected at 23, 31, 39, 47, 55, 63, 71, 79, 87, 102 and 145 DAPs. Twenty fruits with relatively uniform sizes were collected at each time point, refrigerated, and transferred to the laboratory for further study.

### 4.2. Determination of Stone Cell Content

Stone cell content was measured as described by Cai [20]. Pulp (5 g) from between 2 mm beneath the peel to 0.5 mm from the core was collected and stored at −20 °C for 24 h and then homogenized at 20,000 rpm for 3 min. Homogenized pulp was incubated in water, then the upper suspension was decanted, and the process was repeated several times until the upper liquid was clear. Stone cells were oven-dried and weighed, and the content was calculated as follows: (stone cell content (%) = weight of stone cell (g)/weight of pulp (g) × 100). Three biological replicates were analysed for each group.

### 4.3. Determination of Lignin Content

The lignin contents of samples were analysed according to Syros [30] with some modification. A small amount (0.02 g) of dried powder from the pear pulp was placed into the grinding mouth tube. Then, 2 mL of 25% (*v*/*v*) acetyl bromide in acetic acid and 0.1 mL of perchloric acid were added, and the mixture was *heated by a water* *bath at* 65 °C for 30 min with shocking every 10 min to dissolve the powder. Then, 3 mL of sodium hydroxide (2 mol/L) was added, and the mixture was transferred to a centrifuge tube and centrifuged at 6000× *g* for 10 min to collect the supernatant. The volume was corrected to 50 mL with acetic acid, and the absorbance at 280 nm was measured. Three biological replicates were analysed for each group.

### 4.4. Protein Extraction

The pear fruits of 47 DAPs were selected for protein extraction according to Isaacson T [31] with some modification. Briefly, the total protein of each sample was extracted by a TCA/acetone method. Samples were centrifuged at 15,000× *g* for 1 min at 4 °C, and then the precipitate was collected and dried by vacuum freeze-drying. The precipitate was resuspended in cold phenol extraction buffer, and the protein was extracted according to the phenol extraction method. The resulting supernatant contained the extracted protein. The concentrated protein extracts were stored at −80 °C for iTRAQ analysis. Three biological replicates of each group were prepared for the proteomic experiments.

### 4.5. Protein Samples Preparation and Labelling

Proteins were digested and labelled using the FASP method as described previously [32]. First, 100 μg of protein from each sample was added into five volumes of cold acetone at −20 °C for 1 h. The samples were then centrifuged to collect the precipitate, which was dried by vacuum freeze-drying. Dissolution buffer was added to the precipitate, and then reducing reagent was added. The solution was incubated at 60 °C for 1 h. Cysteine-blocking reagents were added, and the solution was held at room temperature for 10 min, followed by centrifugation in a 10 kDa ultrafiltration tube to clean the protein solution. After adding dissolution buffer, the mixtures were again centrifuged, and this step was repeated three times. The column was placed in a new tube before the sequencing-grade trypsin (50 ng/μL) was added and incubated at 37 °C for 12 h. The mixture was centrifuged to collect the peptides, and the filter units were transferred to new collection tubes. The dissolution buffers were added, and the tubes were centrifuged again. Then, these two filter solutions were combined.

iTRAQ labelling was performed using iTRAQ 8-plex reagent kits (AB Sciex, Framingham, MA, USA) to conduct three replicates. Each vial of iTRAQ reagent rested to reach room temperature, and then the iTRAQ reagent was centrifuged to the bottom of the tube. Isopropanol was added to each room temperature iTRAQ reagent vial. The 50 μL sample (100 μg peptide) was transferred to a new tube, and the protein solutions of pear fruits were labelled with isotope 113 (DW), 114 (DW), 115 (DW), 116 (DJ), 117 (DJ), and 118 (DJ). The iTRAQ reagent was added and incubated at room temperature for 2 h. Afterwards, 100 μL of water was added to stop the labelling reaction. Each tube was vortexed and then spun to collect the solution. The sample was desiccated in a vacuum freeze-dryer for iTRAQ analysis.

### 4.6. Peptide Fractionation and Quantitative Proteomic Analysis by LC-MS/MS

The enzymatic hydrolysate of protein solution was re-suspended with 110 μL of eluent A. Peptide separation was performed in an Agilent 1200 HPLC, and the column was also purchased from Agilent (Wilmington, DE, USA). The specific parameters were as follows: guard column: analytical protection column core 4.6 × 12.5 mm 5-micron; separation column: narrow-bore 5 m 2.1 × 150 mm; and UV detection wavelengths: 210 nm and 280 nm.

Eluent from the first 0–7 min was discarded, and 1 tube per min was collected from 8 to 52 min. Then, 4–5 tubes were combined for a total of 10 tubes of pear fruit solution, which were thoroughly frozen. The eluents were A water/ACN/FA (98:2:0.1, *v*/*v*/*v*) and B water/ACN/FA (5:95:0.1, *v*/*v*/*v*). The linear gradient was as follows: 2–2% B (3 min), 3–6% B (0.01 min), 6–25% B (36.9 min), 25–38% B (10 min), 38–90% B (0.01 min), 90–90% B (9.99 min), 90–2% B (0.01 min), and 2–2% B (4.99 min) at a constant flow of 0.3 mL/min.

All analyses were performed on a Triple TOF 5600 System (AB SCIEX) fitted with a Nanospray III source (AB SCIEX). Samples were loaded by a capillary C18 trap column (3 cm × 100 µm) and then separated by a C18 column (15 cm × 75 µm) on an Eksigent NanoLC-1D plus system (AB SCIEX,). The flow rate was 300 nL/min, and the linear gradient was 70 min (from 5–35% B in 70 min).

Data were acquired with a 2.5 kV ion spray voltage, 30 PSI curtain gas, 5 PSI nebulizer gas, and 150 °C interface heater temperature. For information-dependent acquisition (IDA), the MS scans were acquired in 250 ms, and the product ion scans that exceeded a threshold of 150 counts per second (counts/s) with a 2 to 5 charge-state were collected at most 35 times. Each cycle time was fixed to 2.5 s. Dynamic exclusion was set as 18 s [33].

### 4.7. Protein Identification and Quantification

The iTRAQ data were processed with Protein Pilot Software v.5.0 (AB Sciex, Framingham, MA, USA) [33] against the Dangshan Su database using the Paragon algorithm. The database was produced from the transcriptome. The experimental data from tandem mass spectrometry (MS) were matched with the transcriptome data to obtain the resulting protein identification. Protein identification was performed with the search option “emphasis on biological modifications”.

Protein identification was performed with the search option “emphasis on biological modifications”. Screening of the reliable proteins was performed using the following parameters: peptide ≥2 and false discovery rate (FDR) >1%. Invalid values and the anti-library data were removed, and differentially expressed proteins were screened based on reliable proteins. The protein solutions of DW were labelled with isotopes 113, 114 and 115, and the protein solutions of DJ were labelled with isotopes 116, 117 and 118. For differential expression analysis, the fold change was calculated as the average ratios of 113/116, 113/117, 113/118, 114/116, 114/117, 114/118, 115/116, 115/117 and 115/118. Proteins with a fold change of >1.2 or <0.83 were considered to be differentially expressed.

### 4.8. Bioinformatics Analysis

First, we homology mapped the identified proteins to *Arabidopsis thaliana* based on sequence similarity and then carried out the follow-up biological function information analysis through comparative analysis. A multi-omics data analysis tool, OmicsBean (http://www.omicsbean.cn), which integrated GO enrichment and KEGG pathway analysis, was employed to analyse the obtained differentially expressed proteins [34]. Analysis of protein-protein interaction was based on the String (http://string.embl.de/) database and Cytoscape (http://www.cytoscape.org/) software. If *p* < 0.05, the GO term or pathway was regarded as a significant enrichment of differential proteins. Using this method, we determined the main biological function or pathway of differential proteins by GO or KEGG pathway enrichment analysis of Dangshan Su fruit that had been pollinated with different varieties [34].

### 4.9. qRT-PCR Analysis of the Differentially Expressed Proteins

To validate the expression patterns exposed by transcript abundance measurement, three candidate genes that were related to the synthesis of lignin and phenylpropanoid biosynthesis were further analysed by quantitative real-time PCR. The total RNA was extracted from the collected samples using a total RNA isolation kit (Tiangen, Beijing, China). The DNase-treated RNA was reverse transcribed using a PrimeScript RT Reagent Kit with gDNA Eraser (Perfect Real Time, Takara, Dalian, China) [35]. The *tubulin* gene (ID: AB239680.1) was used as an internal reference [36]. The gene-specific primers (Table 2) were designed to amplify three genes by Beacon Designer 7 software (PREMIER Biosoft, Palo Alto, CA, USA), and the primers for these genes were synthesized by Sangon Biotech, Co., Ltd. (Shanghai, China). qRT-PCR analysis was carried out by a Bio-rad CFX96 Touch^TM^ Deep Well Real-Time PCR Detection Systemusing SYBR^®^ Premix ExTaq^TM^ II (Takara, Tokyo, Japan) based on the manufacturer’s protocol. The qRT-PCR amplification was performed as Cao described [36]. The relative expression levels of these genes were estimated by the 2^−ΔΔCT^ method [37]. The reactions were performed in triplicate, and the results were averaged.

## Figures and Tables

**Table 1:** Differentially expressed proteins involved in lignin formation.

Accession	Protein Name	Gene	Coverage %	Peptides (95%)	Fold Change
MSTRG11809.4	β-Glucosidase 15	*BGLU15*	30.7	8	1.2392
Pbr022326.1	Peroxidase 47	*PER47*	14	2	0.7949
Pbr035962.1	Laccase-4	*IRX12*	10.6	2	0.8231

MSTRG: novel; Pbr: known.

**Table 2:** Primers used in qRT-PCR.

Gene ID	Forward Primer (5′–3′)	Reverse Primer (5′–3′)
Tubulin	TGGGCTTTGCTCCTCTTAC	CCTTCGTGCTCATCTTACC
MSTRG11809.4	TCAACTTCACTCCTTCTGATT	CACGCACTAATAACTTATACAACA
Pbr022326.1	GTAAATGGTTATGCCTTCA	TGTTCTTATTTCTCCTTTGG
Pbr035962.1	GTGCACACAACATGGGGGCTTAAGA	GAAGAGATTGATTAGGTCCTTTACC

MSTRG: novel; Pbr: known.

## Supplementary Materials

The following are available online at http://www.mdpi.com/1420-3049/23/3/548/s1. Table S1: The enriched terms by GO hierarchy enriched in biological process of max level 7, Table S2: Differentially expressed proteins involved phenylpropanoid biosynthesis.
